# Comparison of feasibility and effectiveness of tunneled dialysis catheter placement with or without DSA guidance: a propensity score-matched cohort study

**DOI:** 10.1080/0886022X.2024.2376935

**Published:** 2024-07-09

**Authors:** Yiwei Shang, Shujun Pan, Chen Jin, Danna Zheng, Xiujun Xu, Bin Zhu, Li Zhao, Juan Jin, Qiang He, Xiaogang Shen

**Affiliations:** a Department of Nephrology, Urology and Nephrology Center, Zhejiang Provincial People’s Hospital, Affiliated People’s Hospital, Hangzhou Medical College, Hangzhou, Zhejiang, China; b Clinical School of Medicine, Hangzhou Normal University, Hangzhou, Zhejiang, China; c Department of Nephrology, The First Affiliated Hospital of Zhejiang Chinese Medical University, Zhejiang Provincial Hospital of Traditional Chinese Medicine, Hangzhou, Zhejiang, China

**Keywords:** Catheters, angiography, digital subtraction angiography, ultrasonography, renal dialysis

## Abstract

**Background:**

In some resource-limited regions, the placement of tunneled dialysis catheters (TDC) is often preferred under ultrasound guidance rather than fluoroscopy. This study compared ultrasound-and digital subtraction angiography-guided (DSA)-guided TDC in renal replacement therapy.

**Methods:**

This retrospective cohort study included all TDC placements performed at our hospital between January 2020 and October 2022. We utilized 1:1 propensity score matching (PSM) to balance the demographic and clinical characteristics of the DSA-guided and ultrasound-guided groups. Dialysis prescriptions and actual dialysis completion were assessed using intraclass correlation coefficients (ICC). Multivariable logistic regression analyses determined the risk factors for early termination of dialysis. The differences in adverse events, catheter function, and catheter tip position were evaluated between the two groups.

**Results:**

The study included 261 patients (142 in the DSA-guided group and 119 in the ultrasound-guided group). After PSM, 91 patients were included in each group, with no significant baseline differences (*p* > .1). Both groups achieved adequate catheter blood flow and ultrafiltration volumes without deviations from dialysis prescriptions (ICC ≥ 0.75). The DSA-guided group had fewer early dialysis terminations than the ultrasound-guided group (3.3 *vs.* 12.0%, *p* = .026). The position of the catheter tip in the right atrium was more consistent in the DSA-guided group (100 *vs.* 74.2%, *p* < .001).

**Conclusion:**

Hemodialysis catheters inserted under DSA guidance exhibited superior performance compared to those inserted under ultrasound guidance, primarily due to more accurate catheter tip positioning. DSA guidance is recommended when ensuring optimal catheter tip placement.

## Background

Chronic kidney disease (CKD) is a prevalent global health issue affecting an estimated 9.1% of the population worldwide [1]. Renal replacement therapy (RRT) is a crucial aspect of managing CKD [2]. The anticipated rise in the number of individuals requiring RRT, projected to affect ∼5.4 million people globally by 2030 [3], underscores the increasing ­significance of efficient and safe vascular access for hemodialysis. Tunneled hemodialysis catheters (TDCs) provide a medium-to-long-term vascular access option for patients with end-stage kidney disease and lower risk of infectious complications [4]. TDCs are often the best and safest option for patients with limited vascular access or recurrent arteriovenous access issues [5]. Currently, TDCs are widely used in patients requiring RRT, particularly in cases of arteriovenous access thrombosis and maturation failure or delay [6].

Placement techniques for TDC insertion vary across different areas. The KDOQI 2019 guidelines recommend the use of imaging guidance for TDC insertion to ensure safe and accurate catheter tip positioning [7]. Thus, TDC insertion using fluoroscopy is often the preferred choice in developed countries. However, the application of fluoroscopy is limited by high equipment requirements, high procedure costs, and radiation exposure. Even in developed countries, such as Hungary, the availability of fluoroscopy can be limited due to these factors and the need for dedicated radiology personnel [8]. In some resource-limited areas, ultrasound guidance is the only available technique, owing to the unavailability of DSA and other advanced methods. In China, most TDC insertions are performed under ultrasonographic guidance, considering these limitations. In large hospitals with access to digital subtraction angiography (DSA) equipment and qualified personnel, the DSA-guided TDC placement technique is used for a subset of patients.

Currently, the feasibility and effectiveness of ultrasound-guided TDC insertion compared to fluoroscopy-guided TDC insertion remain unclear. Several studies have compared the outcomes of TDC insertion with and without fluoroscopy [9,10]. However, these studies mainly focused on the success rate of surgery, complications, and long-term postoperative patency of the catheter, with little emphasis on immediate catheter usage, the major goal of hemodialysis catheter placement. Additionally, it is uncertain whether these findings in developed countries can be generalized to regions with limited medical resources owing to differences in ultrasound and fluoroscopy techniques. This study compared the outcomes of the first post-surgical hemodialysis session between ultrasound-guided and DSA-guided TDC placement to provide valuable insights into the selection of a more cost-effective approach without compromising patient safety and treatment outcomes.

## Materials and methods

### Study design and data collection

This was a retrospective cohort study involving all TDC insertions performed at our hospital between January 2020 and October 2022. Patient demographics, laboratory results, surgical details, and hemodialysis information were extracted from electronic medical records. The study adhered to the Strengthening the Reporting of Observational Studies in Epidemiology guidelines. This study was approved by the Institutional Ethics Committee of Zhejiang Provincial People’s Hospital (Ethical Approval Number: QT2024052). The requirement for informed consent was waived by the committee due to the observational and retrospective nature of the study.

### Participants

The inclusion criteria were as follows: age >18 years, availability of complete medical records including preoperative and postoperative data, and confirmation of venous patency through bedside ultrasound assessment at the catheter insertion site. Participants should not have used systemic anticoagulation within 24 h before scheduled TDC placement, should have negative blood cultures, and should not have had any localized infection at the intended TDC insertion site within 24 h of planned insertion. The exclusion criteria for this study were as follows: a history of occlusive/stenotic disease of the veins, hemodynamic instability (defined as a systolic blood pressure <90 mmHg or mean arterial pressure [MAP] < 70 mmHg, a decrease in systolic blood pressure >40 mmHg from baseline, or an increased lactate concentration >4 mmol/L), a history of severe cardiovascular or hematologic disease that could potentially affect the outcomes of the study and a history of kidney transplantation.

Propensity scores were calculated using logistic regression, considering the aforementioned demographic and clinical characteristics. The 1:1 nearest neighbor matching with a caliper of 0.3 was conducted using the R package MatchIt. The distance between each unit in one group and units in the other group was computed. Each unit was matched with a control unit. The matching process was ‘greedy’, meaning that no action was taken to optimize an overall criterion; each match was selected without considering the other matches that might occur subsequently. The balance between the two groups was assessed using standardized mean differences (SMD). An SMD of 0.1 or less indicates an ideal balance, while an SMD of 0.2 or less is considered an acceptable balance [11].

### Endpoints and definitions

This study evaluated various outcome measures, including adverse events associated with surgery (kinked catheter, arrhythmias, pneumothorax, hemothorax, venous air embolism, infection, and surgical site bleeding), catheter tip position, and adverse events related to the first postoperative hemodialysis session (such as events leading to early termination of dialysis, air alarm, and blood coagulation). Additional parameters included prescribed and actual blood flow, and ultrafiltration during the first postoperative hemodialysis session. Infections were classified as exit-site infections, bacteremia, and presumed catheter-associated infections. Bleeding was defined as any blood-related event requiring additional sterilization and dressing after surgery or during hemodialysis. Catheterization history referred to the previous placement of a hemodialysis catheter at the same site. Hypotension was defined as systolic blood pressure <90 mmHg or diastolic blood pressure <60 mmHg monitored during dialysis. Blood coagulation was determined by the hemodialysis operator at the end of dialysis and recorded electronically. Blood flow and ultrafiltration volume were automatically recorded using a hemodialysis machine.

**Figure 1.**
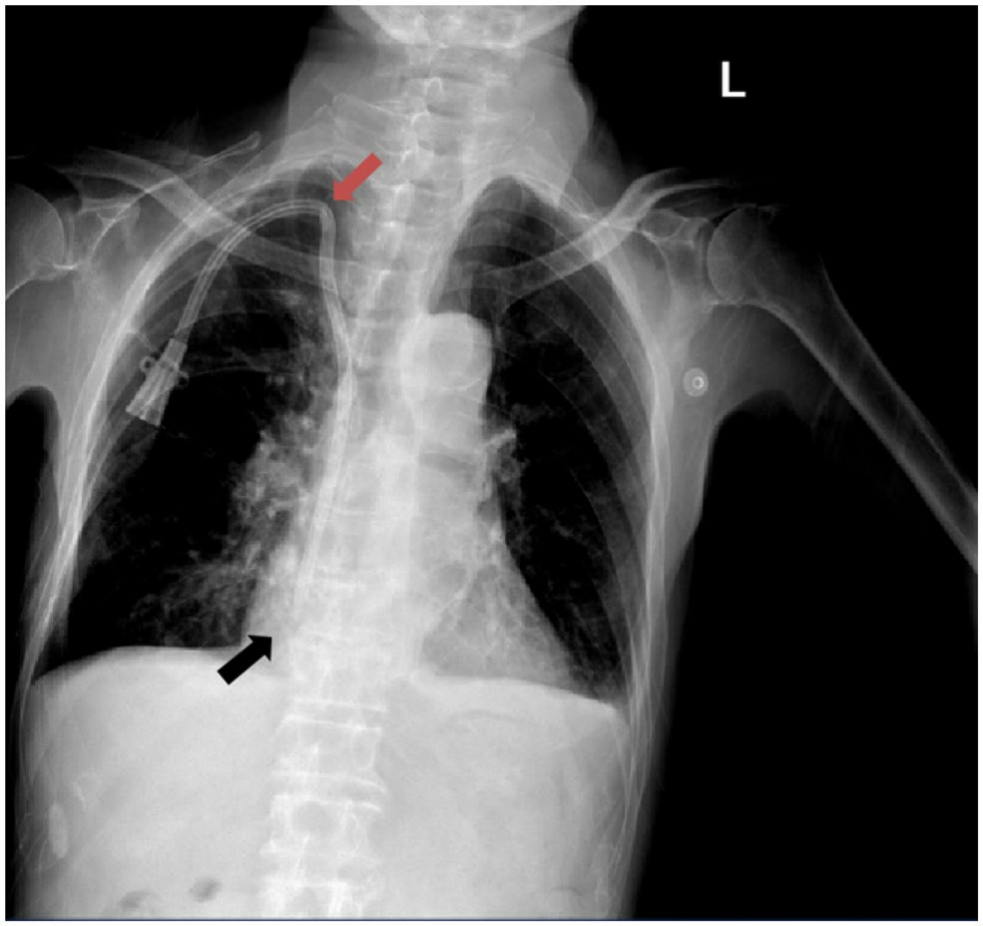
Ultrasound-guided group required postoperative chest X-rays for review. Red arrows indicate the occurrence of kinked catheter events, while the black arrows indicate the position of the catheter tip.

To confirm the position of the catheter tip, video playback was used for DSA-guided procedures, while postoperative chest radiographs were relied upon for ultrasound-guided procedures (Figure 1). The most commonly used catheters were palindromic catheters (Covidien) and Bard Hemosplit catheters (Bard), chosen by patients based on their financial status. The cost of contrast agents used during DSA-guided surgery has changed owing to China’s centralized procurement policy, decreasing from 316.47 to 84.53 CHY, as of October 2021. This study was based on the latest pricing.

According to the KDOQI Clinical Practice Guidelines for Vascular Access and the Chinese Expert Consensus on Vascular Access for Hemodialysis, the optimal position for the catheter tip of a TDC placed in the right internal jugular vein is the mid-upper part of the right atrium [7].

### Bias

We employed PSM to balance baseline characteristics and reduce selection bias. Additionally, we conducted multivariate logistic regression analysis focusing on a specific outcome measure where the two groups differed. This analysis helped to quantify the effect of each technique by adjusting for confounders to ensure reliable conclusions.

### Surgical technique

All surgeries in this study were performed by two senior doctors with over 10 years of surgical experience. In accordance with the emerging global trend toward shared decision-making [12], our approach involved detailed discussions with patients about the available procedural options. Ultrasound-guided procedures are typically performed in hemodialysis units, providing a quicker, albeit less precise, option suitable for urgent needs. Conversely, the more precise DSA-guided procedures with higher precision were conducted in a specialized DSA department. Owing to the limited availability of operating rooms in the DSA department, which often prioritizes cardiac catheterization procedures, there were delays in scheduling DSA-guided surgeries. Therefore, the choice between ultrasound and DSA guidance was based not only on medical criteria but also on a collaborative decision-making process with the patients, considering the urgency of their dialysis treatment and resource availability.

First, local anesthesia was administered at the puncture site to ensure patient comfort and minimize pain. A needle was inserted into the target vessel under ultrasound guidance. Next, a guidewire was advanced through the needle and into the vessel, providing a pathway for subsequent steps. A small incision was made at the insertion site, and the subcutaneous tissue was dilated using a dilator. A catheter was carefully inserted into the vessel over the guidewire and secured using sutures. The main difference between the procedures with and without DSA guidance was the inclusion of a DSA machine and contrast supplies. Under DSA guidance, the procedure can be conducted using real-time visualization, ensuring accurate placement of the catheter tip in the right atrium. In contrast, ultrasound-guided procedures relied on postprocedural chest radiography to confirm the position of the catheter tip.

Based on this, the study objectives were divided into two groups: DSA-guided- and ultrasound-guided.

### Cost of procedures using DSA-guidance and ultrasound-guidance

Cost analysis revealed increased expenses for patients using DSA compared to ultrasound alone (total charges were ￥5783.53 for Palindrome or ￥4433.53 for Bard *vs.* ￥4185.00 for Palindrome or ￥2835.00 for Bard). The bulk of the total cost was the hemodialysis catheter. Excluding the catheter cost, the DSA-guided procedure cost ￥2228.53 compared to ￥630.00 for the ultrasound-guided procedure (Table S1). Thus, excluding the hemodialysis catheter cost, the total cost of the ultrasound-guided procedure was only 28.27% of the total cost of the DSA-guided procedure.

### Statistical analysis

Data that followed a normal distribution are represented as mean ± standard deviation (*SD*), and differences between groups were compared using Student’s *t*-test. For variables that did not follow a normal distribution, the median with interquartile range (IQR) was presented, and the Mann–Whitney *U* test was used to assess the differences. Categorical data were compared using either the chi-square test or Fisher’s exact probability test, depending on appropriateness. Dialysis prescriptions and actual dialysis completion were assessed using ICC, with the following interpretation guidelines: <0.40 (poor), 0.40–0.59 (fair), 0.60–0.74 (good), and 0.75–1.0 (excellent) [13].

Logistic regression was used for multivariate analysis. To determine which variables to include in the model, a subset of variables (age, catheterization technique, catheterization site, sex, catheter type, dialysis history, scheduled dialysis hours, BMI, C-reactive protein (CRP), anticoagulant use, and catheterization history) was selected based on *p*-values from the univariate analysis, as well as previously published literature, researchers’ knowledge, and experience. This approach allowed the inclusion of clinically relevant factors, even if they did not reach statistical significance in the univariate analysis.

All statistical analyses were performed using R software, version 4.2.2, and SPSS statistics, version 26.0. A *p*-value <.05 was considered statistically significant.

## Results

### Baseline patient characteristics

Of the 261 patients enrolled in this study, 142 underwent DSA-guided Surgery, and 119 underwent ultrasound-guided surgery. Before PSM, the DSA-guided group had significantly older patients (*p* = .01), catheterization history (*p* = .003), blood flow rate (*p* = .03), and scheduled dialysis time (*p* = .009) compared to the ultrasound-guided group. Precisely 9.9% of patients in the DSA-guided group opted for a Bard catheter, compared to 21.8% in the ultrasound-guided group (*p* = .01). We balanced the demographics, laboratory results, surgical information, and hemodialysis information of the two groups using PSM. After 1:1 propensity matching, the SMD of all baseline characteristics was <0.2, indicating that the two groups (91 patients per group) were generally balanced (Table 1).

**Table 1.** Baseline covariates before and after matching.

Characteristics	Before matching				After matching			
	DSA-guided	Ultrasound-guided	*p*	SMD	DSA-guided	Ultrasound-guided	*p*	SMD
*n*	142	119			91	91		
Age (years)			.01	−0.297			.99	0.002
Mean ± *SD*	68 ± 13	63 ± 17			65 ± 13	65 ± 15		
Median (IQR)	70 (59, 78)	64 (53, 77)			66 (56, 74)	67 (57, 77)		
Gender (%)			.32				.75	
Male	100 (70.4%)	77 (64.7%)		−0.120	26 (28.6%)	28 (30.8%)		0.046
Female	42 (29.6%)	42 (35.3%)		0.120	65 (71.4%)	63 (69.2%)		−0.046
BMI (kg/m^2^)			.50	0.085			.79	−0.039
Mean ± *SD*	23.4 ± 3.9	23.7 ± 3.7			23.7 ± 3.8	23.6 ± 3.6		
Median (IQR)	22.9 (21.1, 25.2)	23.5 (21.6, 26.1)			23.0 (21.5, 26.1)	23.4 (21.7, 25.9)		
Platelet (10^9^/L)			.56	−0.072			.65	−0.066
Mean ± *SD*	182 ± 80	176 ± 80			186 ± 78	181 ± 76		
Median (IQR)	170 (134, 227)	167 (123, 213)			175 (139, 230)	179 (133, 225)		
CRP (mg/L)			.96	0.006			.68	−0.059
Mean ± *SD*	27 ± 32	27 ± 41			32 ± 35	29 ± 45		
Median (IQR)	14 (6, 37)	10 (3, 34)			15 (6, 50)	10 (3, 36)		
APPT (S)			.61	−0.080			.51	0.093
Mean ± *SD*	30.2 ± 8.9	29.7 ± 5.9			29.6 ± 5.4	30.1 ± 5.8		
Median (IQR)	28.5 (26.8, 32.4)	28.6 (25.9, 31.2)			28.3 (26.9, 32.5)	29.2 (26.8, 31.7)		
Fibrinogen (g/L)			.93	−0.012			.50	−0.102
Mean ± SD	4.25 ± 1.45	4.24 ± 1.39			4.48 ± 1.43	4.34 ± 1.43		
Median (IQR)	4.12 (3.07, 5.20)	4.20 (3.27, 5.09)			4.39 (3.41, 5.41)	4.29 (3.54, 5.19)		
Dialysis history (%)			.08				.96	
Hemodialysis	40 (28.2%)	20 (16.8%)		−0.304	17 (18.7%)	17 (18.7%)		0.000
Peritoneal dialysis	7 (4.9%)	9 (7.6%)		0.100	7 (7.7%)	6 (6.6%)		−0.042
Primary dialysis	95 (66.9%)	90 (75.6%)		0.203	67 (73.6%)	68 (74.7%)		0.026
Catheterization history (%)			.003				>.99	
Yes	13 (9.2%)	1 (0.8%)		0.911	0 (0.0%)	1 (1.1%)		0.120
No	129 (90.8%)	118 (99.2%)		−0.911	91 (100.0%)	90 (98.9%)		−0.120
Catheterization site (%)			.11				.85	
Right femoral vein	19 (13.4%)	28 (23.5%)		0.239	16 (17.6%)	19 (20.9%)		0.078
Right internal jugular vein	120 (84.5%)	89 (74.8%)		−0.224	74 (81.3%)	71 (78.0%)		−0.076
Left femoral vein	2 (1.4%)	2 (1.7%)		0.021	1 (1.1%)	1 (1.1%)		0.000
Left internal jugular vein	1 (0.7%)	0 (0.0%)		−0.114	0 (0.0%)	0 (0.0%)		0.000
Catheter type			.007				.23	
Bard	14 (9.9%)	26 (21.8%)		0.290	12 (13.2%)	18 (19.8%)		0.160
Palindrome	128 (90.1%)	93 (78.2%)		−0.290	79 (86.8%)	73 (80.2%)		−0.160
Time to first postoperative use of TDC (days)			.52	−0.085			.47	−0.127
Mean ± *SD*	1.82 ± 2.98	1.60 ± 2.68			2.10 ± 3.42	1.76 ± 2.88		
Median (IQR)	1.00 (0.00, 2.00)	1.00 (0.00, 2.00)			1.00 (0.00, 2.00)	1.00 (0.00, 2.00)		
Scheduled dialysis time (h)			.009	−0.316			.67	−0.058
Mean ± *SD*	3.43 ± 0.76	3.16 ± 0.83			3.34 ± 0.71	3.29 ± 0.80		
Median (IQR)	3.65 (3.00, 4.00)	3.00 (2.50, 4.00)			3.00 (3.00, 4.00)	3.60 (2.80, 4.00)		
Blood flow rate prescription (ml/min)			.03	−0.281			.80	−0.036
Mean ± *SD*	219 ± 27	212 ± 26			215 ± 25	214 ± 25		
Median (IQR)	220 (200, 250)	200 (200, 235)			200 (200, 230)	200 (200, 245)		
Ultrafiltration volume prescription (ml)			.32	−0.122			.92	0.015
Mean ± *SD*	1,315 ± 795	1,215 ± 818			1,233 ± 797	1,245 ± 867		
Median (IQR)	1,200 (600, 1,900)	1,000 (500, 1,700)			1,000 (550, 1,800)	1,000 (500, 1,800)		
Hemodialysis mode (%)			.08				.77	
HD	105 (73.9%)	83 (69.7%)		−0.091	65 (71.4%)	67 (73.6%)		0.048
HF	33 (23.2%)	34 (28.6%)		0.118	23 (25.3%)	23 (25.3%)		0.000
HDF	4 (2.8%)	2 (1.7%)		−0.088	3 (3.3%)	1 (1.1%)		−0.171
Anticoagulant (%)			.64				.86	
Heparin	37 (26.1%)	32 (26.9%)		0.019	24 (26.4%)	24 (26.4%)		0.000
No anticoagulant	49 (34.5%)	49 (41.2%)		0.136	34 (37.4%)	35 (38.5%)		0.022
Low molecular weight heparin	47 (33.1%)	31 (26.1%)		−0.161	28 (30.8%)	25 (27.5%)		−0.075
Sodium citrate	8 (5.6%)	7 (5.9%)		0.011	5 (5.5%)	7 (7.7%)		0.093
Fondaparinux Sodium	1 (0.7%)	0 (0.0%)		−0.114	0 (0.0%)	0 (0.0%)		0.000

### Catheter function following TDC placement

**Figure 2.**
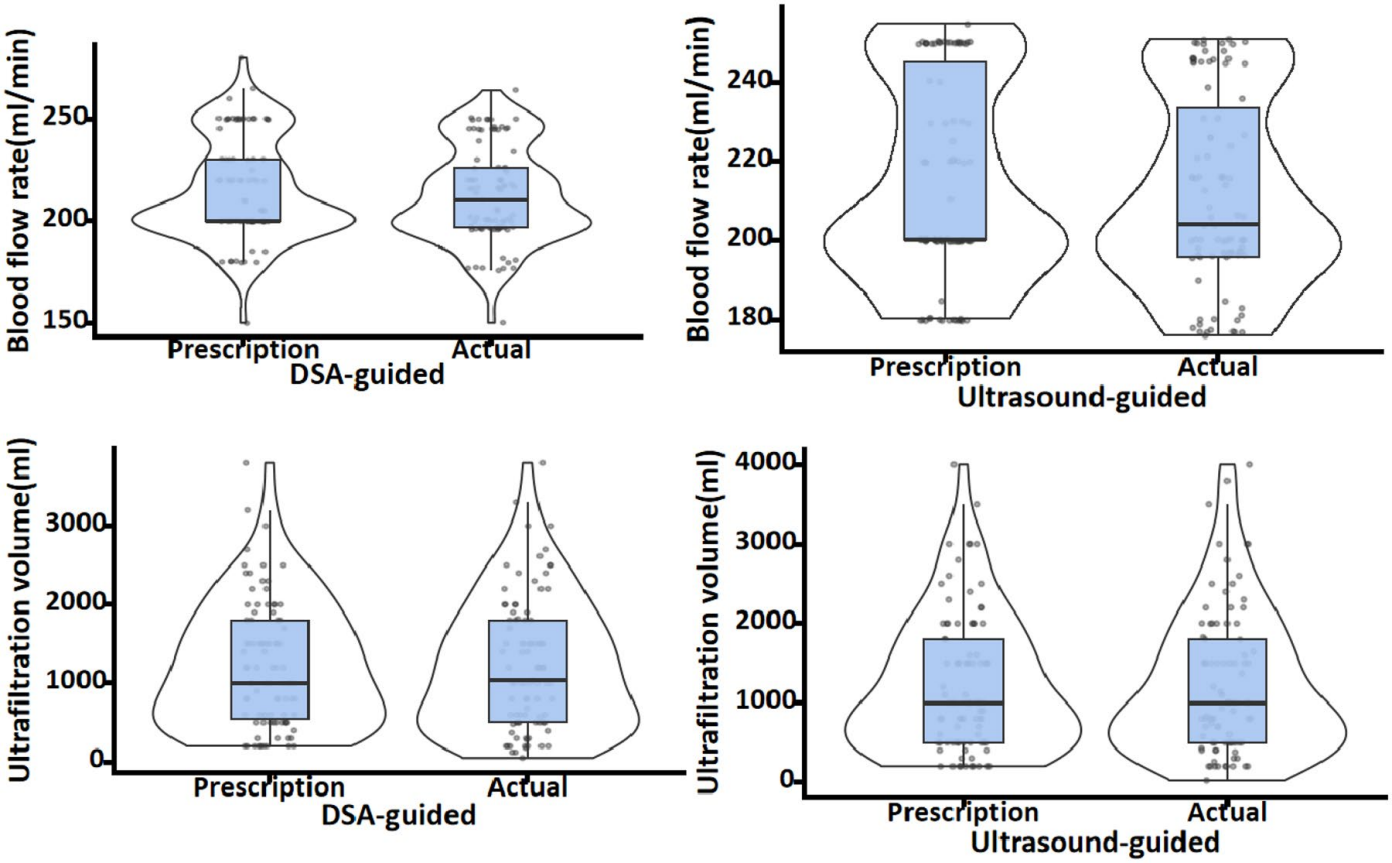
Violin plot of blood flow rate and ultrafiltration volume during the initial postoperative hemodialysis session.

The functional outcomes of the catheters in both groups are presented in Table 2. In the DSA-guided group, the median catheter blood flow was 210 (IQR 197–226) mL/min and the ultrafiltration volume was 1040 (IQR 500–1800) mL. The ICCs for dialysis prescriptions were 0.762 (95% CI: 0.660–0.837) and 0.959 (95% CI: 0.939–0.973), respectively. In the Ultrasound-guided group, a median catheter blood flow of 204 (IQR 196–233) mL/min and an ultrafiltration volume of 1000 (IQR 500–1798) mL were achieved. The ICCs for dialysis prescriptions were 0.806 (95% CI: 0.720–0.868) and 0.982 (95% CI: 0.973–0.988), respectively (Figure 2). There were no significant deviations in blood flow or ultrafiltration volume from the dialysis prescription in either group.

**Table 2.** Catheter function following TDC placement.

Variables	DSA-guided		Ultrasound-guided	
	Median (IQR)	ICC(95%CI)	Median (IQR)	ICC(95%CI)
Blood flow rate (ml/min)		0.762 (0.660–0.837)		0.806 (0.720–0.868)
Prescription	200 (200–230)		200 (200–245)	
Actual	210 (197–226)		204 (196–233)	
Ultrafiltration volume (ml)		0.959 (0.939–0.973)		0.982 (0.973–0.988)
Prescription	1000 (550–1800)		1000 (500–1800)	
Actual	1040 (500–1800)		1000 (500–1798)	

### Adverse events during surgery and hemodialysis

No serious complications related to catheter placement, such as venous air embolism, arrhythmias, pneumothorax, or hemothorax, were observed in any patient in either group. There were slightly more infections (*p* = .50) and bleeding events (*p* = .18) in the DSA-guided group; however, the difference was not statistically significant. Interestingly, the ultrasound-guided group experienced a kinked catheter event that required a second procedure, which was not observed in the DSA-guided group (Figure 2). The occurrences of air alarm events (*p* > .99), blood coagulation events (*p* = .86), and hypotension during dialysis (*p* = .82) did not differ significantly between the two groups. However, the DSA-guided group had significantly fewer events that led to early termination of dialysis compared to the ultrasound-guided group, both before (*p* = .04) and after (*p* = .03) PSM (Table 3). In the DSA group, two events that led to early termination of dialysis were due to severe coagulation of the dialysis line identified during hemodialysis, and one event was due to elevated transmembrane pressure (TMP). In the ultrasound group, six of the 11 events that led to early termination of dialysis were due to severe coagulation in the dialysis line. Additionally, four events were caused by severely elevated venous pressure alarms on the hemodialysis machine, and one event was caused by an air alarm on the machine.

**Table 3.** Adverse events during surgery and hemodialysis.

Variables	Before matching				After matching			
	DSA-guided (*n* = 142)	Ultrasound-guided (*N* = 119)	Risk difference, RD (%) (95% CI)	*p*	DSA-guided (*n* = 93)	Ultrasound-guided (*n* = 93)	Risk difference, RD (%) (95% CI)	*p*
Adverse events associated with surgery								
Kinked catheter	0 (0%)	1 (0.8%)	−0.84 (−2.48 to 0.80)	.46	0 (0%)	1 (1.1%)	−1.10 (−3.24 to 1.04)	>.99
Arrhythmias	0 (0%)	0 (0%)	0	>.99	0 (0%)	0 (0%)	0	>.99
Pneumothorax/Hemothorax	0 (0%)	0 (0%)	0	>.99	0 (0%)	0 (0%)	0	>.99
Venous air embolism	0 (0%)	0 (0%)	0	>.99	0 (0%)	0 (0%)	0	>.99
infection	2 (1.4%)	1 (0.8%)	0.57 (−1.97 to 3.11)	>.99	2 (2.2%)	0 (0%)	2.20 (−0.81 to 5.21)	.50
Surgical site bleeding	14 (9.9%)	8 (6.7%)	3.14 (−3.52 to 9.79)	.36	10 (11%)	5 (5.5%)	−5.49 (−13.45 to 2.46)	.18
Adverse events associated with hemodialysis								
Events leading to early termination of dialysis	7 (4.9%)	14 (11.8%)	−6.84 (−13.63 to −0.04)	.04	3 (3.3%)	11 (12%)	−8.79 (−16.43 to −1.)	.026
Air alarm	1 (0.7%)	1 (0.8%)	0	>.99	0 (0%)	1 (1.1%)	1.10 (−1.04 to 3.24)	>.99
Blood coagulation	41 (28.9%)	39 (32.8%)	−3.90 (−15. to 7.36)	.50	22 (24%)	23 (25%)	−1.10 (−13.63 to 11.44)	.86
Hypotension	23 (16%)	14 (12%)	4.43 (−3.95 to 12.81)	.31	11 (12%)	10 (11%)	1.10 (−8.18 to 10.38)	.82

### Risk factors for early termination of dialysis

**Figure 3.**
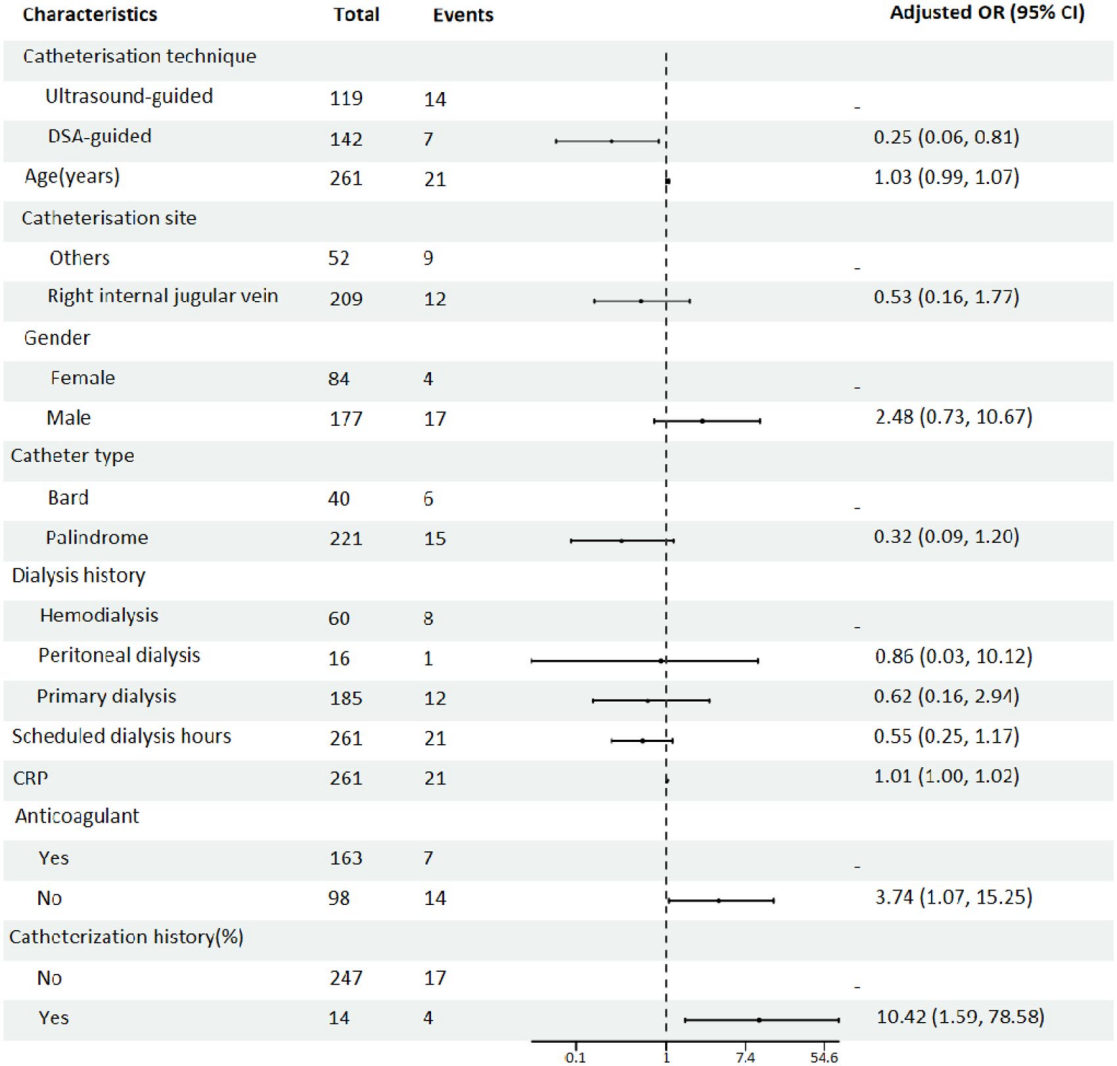
Multifactorial logistic regression Forest plot of risk factors for early dialysis termination.

Univariate logistic regression analyses before PSM demonstrated that catheterization technique (*p* = .05), CRP level (*p* = .009), catheterization history (*p* = .009), catheterization site (*p* = .009), and anticoagulant use (*p* = .007) were significant risk factors for early termination of dialysis (Table 4). Multivariate logistic regression analysis showed that the catheterization technique (*p* = .024), catheterization history (*p* = .009), and anticoagulant use (*p* = .049) were independent risk factors for early termination of dialysis (Figure 3).

**Table 4.** Univariate and multivariate analysis for early termination of dialysis.

Characteristic	Univariable					Multivariable				
	*N*	Event *N*	OR	95% CI	*p*	*N*	Event *N*	OR	95% CI	*p*
Age (years)	261	21	1.03	1.00, 1.06	.11	261	21	1.04	1.00, 1.07	.08
Gender (male), *n* (%)										
Male	177	17	—	—		84	4	—	—	
Female	84	4	0.47	0.13, 1.32	.19	177	17	2.48	0.73, 10.67	.18
BMI (kg/m^2^)	261	21	0.98	0.87, 1.10	.79					
Dialysis history										
Hemodialysis	60	8	—	—		60	8	—	—	
Peritoneal dialysis	16	1	0.43	0.05, 3.75	.45	16	1	0.86	0.03, 10.12	.91
Primary dialysis	185	12	0.45	0.17, 1.16	.10	185	12	0.62	0.16, 2.94	.52
Catheterization history (%)										
No	247	17	—	—		247	17	—	—	
Yes	14	4	5.41	1.54, 19.08	.009	14	4	10.42	1.59, 78.58	.02
Hemoglobin (g/L)	261	21	1.00	0.98, 1.03	.69					
Platelet (×10^9^/L)	261	21	1.00	1.00, 1.01	.76					
CRP	261	21	1.01	1.00, 1.02	.009	261	21	1.01	1.00, 1.02	.08
SIRI (×10^9^/L)	261	21	1.03	0.99, 1.07	.13					
Albumin (g/L)	261	21	0.99	0.92, 1.06	.76					
ALT (U/L)	261	21	1.00	1.00, 1.00	.89					
Creatinine (μmoI/L)	261	21	1.00	1.00, 1.00	.66					
APTT (s)	261	21	1.03	0.99, 1.07	.18					
Fibrinogen (g/L)	261	21	0.85	0.61, 1.19	.34					
Catheterization site										
Others	52	9	—	—		52	9	—	—	
Right internal jugular vein	209	12	0.29	0.12, 0.75	0.009	209	12	0.53	0.16, 1.77	.29
Catheterization technique										
DSA-guided	142	7	—	—		119	14	—	—	
Ultrasound-guided	119	14	2.57	1.00, 6.60	.05	142	7	0.25	0.06, 0.81	.03
Catheter type										
Bard	40	6	—	—		40	6	—	—	
Palindrome	221	15	0.41	0.15, 1.14	.09	221	15	0.32	0.09, 1.20	.08
Anticoagulant										
Yes	163	7	—	—		163	7	—	—	
No	98	14	3.71	1.44, 9.56	.007	98	14	3.74	1.07, 15.25	.049
Blood flow rate prescription (ml/min)	261	21	1.01	0.99, 1.02	.52					
Ultrafiltration volume prescription (ml)	261	21	1.00	1.00, 1.00	.39					
Time to first postoperative use of TDC (days)	261	21	0.95	0.78, 1.14	.57					
Hemodialysis mode										
HD	188	14	—	—						
HF	67	6	1.22	0.45, 3.32	.69					
HDF	6	1	2.49	0.27, 22.77	.42					

### Catheter tip position in the right internal jugular vein

In the DSA-guided group, the position of the catheter tip could be visualized in real-time during surgery. In contrast, in the ultrasound-guided group, confirmation could only be performed through a postoperative review of plain radiographs. After excluding data from missing postoperative radiographs, 74 patients in the DSA-guided group had catheter tips located in the right atrium. In the ultrasound-guided group, 49 out of 66, with the remaining 17 out of 66 patients had catheter tips located in the superior vena cava (Figure 1). Further analysis of the 17 patients with catheter tips in the superior vena cava and the 123 patients with catheter tip positions in the right atrium revealed that patients with catheter tips located in the superior vena cava had a higher incidence of events leading to early termination of dialysis (17.7 *vs.* 3.3%, *p* = .04, RD = 14.4 [95% CI: −4.00 to 32.79]) and blood coagulation (52.9 *vs.* 19.5%, *p* = .005, RD = 33.43 [95% CI: 8.69 to 58.17]) compared to patients with catheter tips located within the right atrium. When the study was limited to patients with catheter tips located in the right atrium, no significant difference was observed between the DSA- and ultrasound-guided groups in terms of events leading to early termination of dialysis (2.7 *vs.* 4.1%, *p* > .99, RD = −1.38 [95% CI: −8.04 to 5.28]).

## Discussion

This study offers a comprehensive comparison between ultrasound- and DSA-guided TDC insertion in patients requiring RRT. The results underscore the importance of accurate catheter tip positioning and its impact on patient outcomes, particularly during the first postoperative hemodialysis session. The similarity in catheter function between the two groups, as evidenced by the ICC for dialysis prescription, indicated that both ultrasound- and DSA-guided TDC placements were effective in achieving the desired blood flow and ultrafiltration volumes. This finding is crucial for clinical practice, as it suggests that despite technological differences, both techniques can provide adequate hemodialysis. However, the study also revealed critical distinctions. The DSA-guided group exhibited fewer incidents, leading to early termination of dialysis. This suggests that real-time visualization afforded by DSA may contribute to more optimal catheter placement, potentially reducing complications, such as severe coagulation and elevated venous pressure alarms during hemodialysis. This aspect is particularly noteworthy because minimizing interruptions in hemodialysis is vital for patient health and efficient treatment.

Both DSA-guided and ultrasound-guided procedures are commonly used for TDC placement. A meta-analysis ­published in the Cochrane Database concluded that ultrasound-guided procedures are superior to blind punctures. However, there is no clear evidence favoring DSA- over ultrasound-guided approaches. Existing literature mainly reports on TDC placement under DSA or ultrasound guidance. For example, studies by Caridi et al. [14] and Sayani et al. [15] have reported the effectiveness and safety of surgery under DSA guidance. Aurshina et al. [16] reported 1065 patients who underwent ultrasound-guided surgeries with no cases of severe complications, such as hemothorax or pneumothorax, with only two cases of arterial injury and three cases requiring postoperative revision due to incorrect catheter placement. Sohail et al. [17] reported 25 cases during the COVID-19 pandemic, where institutions shifted from DSA to ultrasound guidance, achieving a 100% success rate with satisfactory postoperative blood flow and ultrafiltration meeting the requirements of dialysis prescriptions. However, studies directly comparing DSA and ultrasound guidance for hemodialysis catheter placement are lacking. The references for DSA and ultrasound guidance for TDC placement surgery in KDIGO 2019 were sourced from an article published by Yevzlin et al. [18] in 2007, which reported higher surgical success rates and lower costs under DSA guidance. Conversely, studies by Obialo et al. [19] and Chang et al. [9] found that DSA guidance did not confer an advantage over ultrasound guidance and was associated with lower catheter patency rates and higher surgical complications, such as infection and bleeding. However, the lower catheter patency rate in the DSA group did not align with the authors’ clinical knowledge, which may be due to the non-random selection of patients for DSA or ultrasound-guided surgery, with patients opting for DSA surgery having potentially worse baseline conditions. Therefore, in this study, we used PSM to balance as many baseline variables as possible, including demographics, laboratory results, surgical information, and hemodialysis data, to minimize bias. Additionally, the selection of DSA and ultrasound procedures at our institution is primarily based on the availability of the DSA operating room rather than subjective decisions by doctors, significantly reducing selection bias compared with previous studies. Previous studies have mainly focused on the success rate of surgery, complications, and catheter patency after surgery. However, one major goal of placing a hemodialysis catheter is immediate use. Therefore, we included data on the first hemodialysis session post-surgery as the primary outcome measure. Furthermore, previous studies have been conducted in developed countries, but the question of whether surgery guided by DSA is necessary is even more important in developing countries, where medical resources are often limited.

In this study, we compared the effectiveness, safety, and cost of hemodialysis catheter placement using DSA and ultrasound guidance. The results showed that although the DSA guidance group had higher surgical costs, the proportion of catheter tips placed in the correct position was significantly higher than that in the ultrasound-guided group. Additionally, the occurrence of premature dialysis cessation during the first postoperative hemodialysis session was lower in the DSA guidance group. Logistic regression analysis on the occurrence of premature cessation of dialysis identified catheterization technique, catheterization history, and anticoagulant use as independent predictive factors. Furthermore, secondary analysis of patients with different catheter tip positions indicated that the advantages of the DSA group may be due to more accurate catheter tip placement. Some studies have reported that intracavitary ECG can guide catheter tip placement during the procedure [20]. Recent studies have shown that the use of cardiac ultrasound in combination with saline injections can also accurately locate the catheter tip [21,22]. These findings suggest that regardless of the method used, DSA may not be necessary if the surgeon can ensure proper catheter tip placement. Based on the results of the logistic analysis of adverse events leading to premature discontinuation of hemodialysis, we believe that DSA-guided hemodialysis catheter placement is preferable in patients with a history of previous catheter placement and those unable to use anticoagulants during hemodialysis.

DSA devices are not easily accessible in developing countries, and healthcare funding is limited. Even after the introduction of centralized procurement policies for contrast agents used in DSA procedures in China, the costs of DSA-guided surgeries remain significantly higher than those guided by ultrasound [23]. This study revealed, for the first time, the advantages of DSA-guided surgeries, providing a basis for determining when to use DSA procedures appropriately. These findings have several implications for clinical practice. First, they highlight the need for individualized patient care considering both medical and socioeconomic factors. In settings in which DSA is available and affordable, it may be preferred due to its potential to reduce complications and interruptions during dialysis. However, ultrasound-guided TDC placement has emerged as a viable and cost-effective alternative in resource-constrained environments.

## Limitations

Although this study provides valuable insights, it has some limitations. The retrospective nature of the study may introduce bias, and the findings from a single center may not be generalizable to other settings with different patient populations or healthcare infrastructures. Additionally, this study did not explore the long-term outcomes, which are crucial for understanding the durability and overall effectiveness of each technique. Furthermore, due to sample size limitations, this study did not conduct in-depth research on catheter placement sites other than the right jugular vein, which requires larger-scale studies for further exploration.

## Conclusion

The performance of hemodialysis catheters inserted under DSA guidance was superior to that of catheters inserted under ultrasound guidance during postoperative blood dialysis. This may be related to the position of the catheter tip. This study recommends the use of DSA when the catheter tip cannot be reliably placed in the right atrium.

## Supplementary Material

Supplemental Material
