# Supplementary material for: Comparison of feasibility and effectiveness of tunneled dialysis catheter placement with or without DSA guidance: a propensity score-matched cohort study
Source: Ren Fail. 2024 Jul 9;46(2):2376935. doi: 10.1080/0886022X.2024.2376935 (PMC11238648; doi:10.1080/0886022X.2024.2376935)
Supplement: Supplemental Material [file IRNF_A_2376935_SM7096.doc]

**Table S1: Cost Comparison of Procedures Using DSA-guidance and Ultrasound-guidance**

| **Catheterization Technique** | **Facility and Physician Costs** | **￥（CNY）** | **Supplies** | **￥（CNY）** | **Total ￥** |
| --- | --- | --- | --- | --- | --- |
| **DSA-guided** | Ultrasound | 50.00 | Hemodialysis catheter | 3555(Palindrome) or 2205(Bard) |  |
|  | Catheter placement surgery | 520.00 | Guide wire | 204.00 |  |
|  | DSA | 1100.00 | Angiographic sheath | 175.00 |  |
|  |  |  | Contrast agents | 84.53 |  |
|  |  |  | High Pressure Contrast Injection Catheter | 95.00 |  |
|  | **Total** | **1670.00** | **Total** | **4113.53(Palindrome) or 2763.53(Bard)** | **5783.53(Palindrome) or 4433.53(Bard)** |
| **Ultrasound-guided** | Ultrasound | 50.00 | Hemodialysis catheter | 3555(Palindrome) or 2205(Bard) |  |
|  | Catheter placement surgery | 520.00 |  |  |  |
|  | Chest x-ray | 60.00 |  |  |  |
|  | **Total** | **630.00** | **Total** | **3555.00(Palindrome) or 2205.00(Bard)** | **4185.00(Palindrome)or 2835.00(Bard)** |
